# Using the future wheel methodology to assess the impact of open science in the transport sector

**DOI:** 10.1038/s41598-023-33102-5

**Published:** 2023-04-12

**Authors:** Anja Fleten Nielsen, Jakob Michelmann, Attila Akac, Kristel Palts, Anne Zilles, Afroditi Anagnostopoulou, Ove Langeland

**Affiliations:** 1 Institute of Transport Economics Norway, Oslo, Norway; 2 VDI/VDE Innovation + Technik GmbH, Berlin, Germany; 3 Hellenic Institute of Transport/Centre for Research and Technology Hellas, Piraeus, Greece; 4 European Aviation Safety Agency, Cologne, Germany; 5 German Aerospace Center, Cologne, Germany

**Keywords:** Climate-change impacts, Climate-change policy, Energy and society, Environmental impact, Socioeconomic scenarios, Sustainability

## Abstract

Open Science enhances information sharing and makes scientific results of transport research more transparent and accessible at all levels and to everyone allowing integrity and reproducibility. However, what future impacts will Open Science have on the societal, environmental and economic development within the transport sector? Using the Future Wheel methodology, we conducted a workshop with transport experts from both industry and academia to answer this question. The main findings of this study point in the direction of previous studies in other fields, in terms of increased innovation, increased efficiency, economic savings, more equality, and increased participation of citizens. In addition, we found several potential transport specific impacts: lower emission, faster travel times, improved traffic safety, increased awareness for transport policies, artificial intelligence improving mobility services. Several potential negative outcomes of Open Science were also identified by the expert group: job loss, new types of risks, increased cost, increased conflicts, time delays, increased inequality and increased energy consumption. If we know the negative outcomes it is much easier to put in place strategies that are sustainable for a broader stakeholder group, which also increase the probability of taking advantage of all the positive impacts of Open Science.

## Introduction

The purpose of this paper is twofold: First to examine how Open Science will impact the future on a social, environmental and economic level in the transport sector and second, to explore how the Futures Wheel methodology can be used as a methodological framework in order to predict future scenarios. The paper is first and foremost a methodological and empirical in nature. This is the first study on Open Science in the transport sector, and hence we try to establish knowledge about both the positive and negative implications of this phenomenon.

Open Science is “a system change allowing for better science through open and collaborative ways of producing and sharing knowledge and data, as early as possible in the research process, and for communicating and sharing results”^1^. The increasing diffusion of Open Science practices around the globe fostered by political decisions, such as the rollout of the Open Science Policy of the European Union, e.g. across Horizon Europe, the 9th research and innovation framework programme (2021–2027)^1^, could cause a multitude of impacts on research organisation, markets, technologies, society and environment. Open Science can enhance information sharing and makes scientific results of transport research more transparent and accessible at all levels and to everyone allowing integrity and reproducibility leading to more efficiency in research^2^. However, there is limited evidence on how Open Science will impact the future in the transport sector. While the demand for sustainable, seamless, secure and safe transport is growing, new technologies are cost-intensive and their acceptance and transferability to other regions or use cases depends on many factors^3^. Learned from many positive and negative lessons, social, organisational and technical innovations should be assessed in advance and during their diffusion in order to minimize threats caused by unknown side effects. One of the most impressive lessons learned has been the leakage of CFC gases from refrigerators causing tremendous effects, such as shrinking the ozone layer. These, and other lessons lead to the evolvement of qualitative and quantitative impact assessment^4^. In order to create a sustainable environment for Open Science within the transport sector we need to know both potential positive and negative effects, and effects that are specific to transport in regard to potential economic, social and environmental changes. Open Science consists of several different concepts (eg.^5–7^), and for this study Open Data (OD), Open Source Software (OSS), Open Access (OA) and Citizen Science (CS) will be in focus.

Open access has been one of the focus areas of Open Science for a long time. This is also one of the areas within OS where there is quite a lot of disagreement on the advantages^8,9^. It is too expensive for any individual or organization to subscribe to all journals^10^, making a lot of the knowledge produced unavailable to both the general public, but also to other researchers. Opening up the access to research, it gives on one hand a broad citation advantage for researchers who publish openly^8,11,12^. Furthermore, it has economic impacts on innovation enterprises and a range of governmental and non-governmental services, potentially saving a lot of resources^8,9^. On a societal level it can also improve citizen science initiatives and levelling the playing field for researchers in developing countries^8^ However, it could also have negative impacts if high-cost options are allowed in an unregulated marked (ibid.). The business model of OA journals, where the authors pay, also invites predatory journals to enter the market^11^, corrupting the publishing system and promoting unethical behaviour among researchers in need for publications^13^. There has been an increase in the share of open access articles in the transport research community the last decade^14^ and open access publishing has been highly prioritized politically in several countries. However, in the transport research community, the few open access journals have a lot of issues related to quality^15^, making it less attractive for researchers to publish. Also, the models for open access publishing created by coalition S, may have implications for the development of Open Access in general—causing non-equal opportunities on a national level, where some countries can pay for publishing openly in high quality journals, while others cannot afford to pay for these journals^15^.

Open data can possibly add a lot of value to the society. One of the main motivations for opening up publicly funded data is to increase return of investment, generate wealth and manage to solve more complex problems^16^. How and what values will be created in the future is impossible to predict, however, there are some value creation that is already possible to point to^17,18^: transparency, democratic control, participation, self-empowerment, improved or new private products and services, innovation, efficiency and effectiveness of services and new knowledge through combining data sources in large volumes. Some innovation examples increasing social value through participation and self-empowerment are^17^:

- Denmark: Findtoilet.dk, a woman created an app that shows people where all the public toilets are, so that people with bladder issues can move more freely.

- The Netherlands: Vervuilingsalarm.nl, an app that shows the air-quality in your vicinity.

- New York: finding where to walk your dog and people who use the same park.

Other social benefits than empowerment of citizens is increased societal participation, collaboration, and inclusion of marginalized groups^19^.

Several studies have estimated the economic value of open data at several tens of billions of Euros annually in the EU alone. In the Netherlands, the Ministry of Education publishes education-related data for reuse, which has in turn reduced the number of questions they receive, reducing workload and costs^19^. Open data is also seen as a driver for economic growth, contributing to innovation and creation of new business models. Businesses re-use data to gather meaningful insights and develop and improve products and services^19,20^. Despite all the positive possibilities of opening up data, there are also barriers related to open data, and negative aspects needs to be addressed more attention^21^. There is a large potential for open data within the transport sector and the importance is increasing. However, open data is possibly more problematic for the transport research sector than other fields due to large data generation outside the traditional research community^15,22^, making it more difficult to access the data.

Since early 2000, OSS has been identified as a promising strategy to support the efficiency and quality of software developments^23^. Specifically, Linux and Apache were successful case studies that revealed the potential of OSS^24^. Many companies adopted OSS due to its adjustability in order to develop customized applications^53^. More recently, OSS has been adopted in several sciences such as engineering^25^, architecture^26^, physics^27^, biology^28^, etc. In this context, latest EC’s strategies focus on the increase of digital transformation schemes in various sectors to achieve a research-friendly environment through the usage of ICT services and existing open-source data infrastructures.

In general, according to the Open Forum Europe organization^29^, OSS is a powerful tool also offering valuable societal and economic benefits to support innovation. OSS can benefit all users with control options, training opportunities, system’s security and stability. On the other hand, OSS initiatives produce problems related to software patents and their regulatory framework. Free Software Foundation Europe^30^ highlights the problematic relationship between OSS tools and current legal framework. Existing regulations at national and international level leave significant blind spots for the exploitation of data production and outputs. More specifically, various legal risks and problems with OSS products patenting and commercialization bring other direct and indirect societal and economic effects on the scientific research communities and general public subsequently.

Ellis & Van Belle^31^ have studied the barriers and adapters that influence the adoption of OSS by SMEs in South Africa. In general, developing countries primarily focus on cost-efficient alternatives to become competitive against the developed markets (Europe, US, China, Japan, etc.). These researchers highlighted several significant impacts of the usage of OSS tools replacing commercial software solutions for text editing (MS Office), geographical and meteorological representation (ArcGIS, etc.) and other similar software categories. Similar findings were highlighted in research from India^32^. Economic impacts are savings from hardware & software cost, cost of ownership (including training schemes and indirect costs for operation and maintenance of software products), social impacts involve complexity on administration and OSS establishment and change management adaptation of employees within an organization. According to Xing^32^ the presence of commercial open source software can lead to the decrease of the software price and profit for proprietary software producer, while the consumer surplus and social welfare will be increased. Interestingly it does not necessarily cause a decline in the market share for proprietary software producer. Environmentally this could have effects on energy savings from the re-use of older computer units (with Linux OS) and other free-open access OSS tools^31^. OSS is still not used a lot in transport research, but there is a large potential: A survey sent out to all transport research institutions in Europe shows increasingly positive attitudes towards OSS, especially for the last 1–2 years^22^.

Citizen science deals with public participation in scientific research, it often results in advancements in scientific research by improving the scientific community’s capacity, as well as increasing the public's understanding of science to some extend^34^. Citizen science is important to increase democracy in science and promoting universal and equitable access to data and information^35^. Economically, Citizen Science can contribute to the design of new solutions through emerging technologies and making transport more efficient^36^. Citizen Science can also utilize creativity of citizens to react to challenges and in monitoring and designing cities. For example, citizen Science can deliver data to decision-making in policy^37^, to e.g. making cities safer^38^. And lastly, on an environmental level Citizen Science can support research on environmental developments or impacts of certain ecologic factors, such as monitoring the ecologic conditions of areas.^39,40^. Citizen science is especially relevant in transport research as interactive maps of mobility are getting more common (Open Maps), together with apps for tracking (bikes etc.). Also, citizen science is very relevant in order to improve transport accessibility^41^.

## Materials and methods

**Figure 1:**
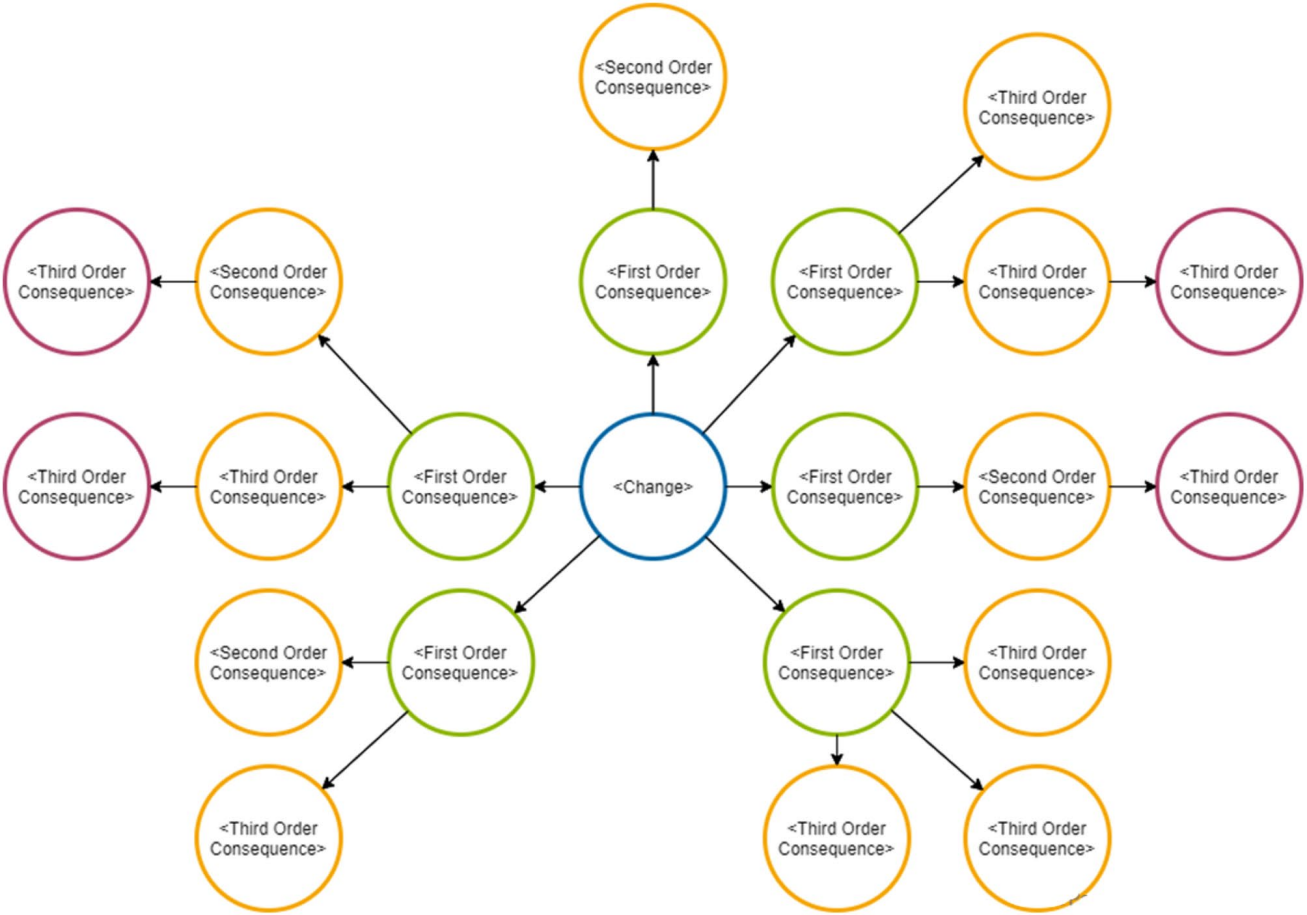
Futures wheel. Source: Visual paradigm online free edition.

The Future Wheel is a methodology used for structuralising brainstorming in order to use it for futuristic analysing of trends impact on society at different levels. It was originally invented by Jerome C. Glenn in 1971^42^, and is used for identifying and packaging primary, secondary, and tertiary consequences of trends (see Fig. 1), events, emerging issues, and future possible decisions^43^. There are also other ways of conducting future research—where the Delphi-method is a common methodology used in transport research in order to predict scenarios of transport use of both people and gods^44–46^. However, this method is very time consuming—and we therefore chose to conduct a somewhat similar method that only require one day of data collection.

We carried out a 2-h digital workshop with experts in different fields of Open Science: Open Data, Open-source software, Citizen Science and Open Access.

The reason for choosing these groups is based on the finding in the BEOPEN project^15,22^:

•Open access: There has been an increase in the share of open access articles the last decade and open access publishing has been highly prioritized politically in several countries. However, in the transport research community open access journals have a lot of issues related to quality. Also, the models for open access publishing created by coalition S1, may have implications for the development of Open Access—causing non-equal opportunities on a country level.

•Open data—There is a large potential for open data and the importance is increasing. Open Data is possibly more problematic for transport than other fields due to large data generation outside the traditional research community.

•Open-source software—OSS is still not used a lot in transport research, but there is a large potential as attitudes are increasingly positive especially in the last 1–2 years.

•Open citizen science—this is especially relevant in transport research as interactive maps of mobility are getting more common (Open Maps), together with apps for tracking (bikes etc.).

A total of 19 European experts were in the workshop representing the following:

- Researchers from universities or transport research institutes
- Transport research associations
- Public transport authorities
- Librarians
- Software engineers

The workshop was recorded in order to analyse the discussion at a later stage.

**Figure 2:**
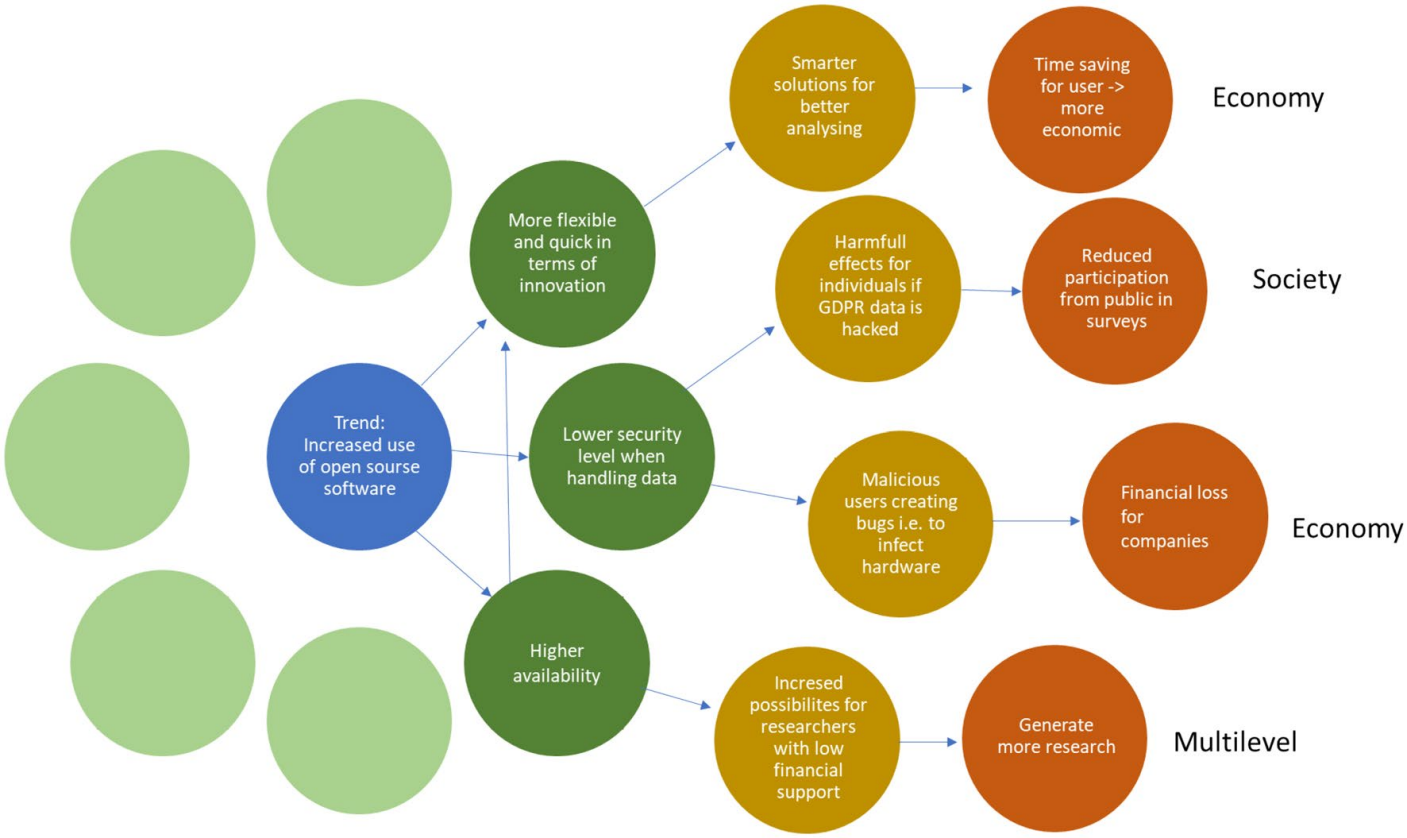
Futures wheel: increased OSS in transport.

There are several different ways of using the future wheel, e.g. panels or desk research, to reach basically similar goals: Identification of, both negative and positive impacts and side-effects of possible changes or trends. This way, potential knowledge gaps on impacts can be reduced. Though, no one can predict complex futures, the application of the methods works as a sparring technique to elaborate a multifaceted picture of possible impacts in the future. On the basis of that, stakeholders can discuss and evaluate wishful, possible or likely impacts in order to work out strategies to support or avoid impacts within foresight processes^42,47,48^. The futures wheel has been proven to derive potential ecologic and socio-economic impacts^49^. In the workshop the methodology based on Glenn^42^ described hereafter has been used. An example for Open Science in transport was provided to make it easier to understand (see Fig. 2 for reference):

1)In the middle of the wheel (Fig. 2) is the trend that you want to analyse: Increased use of open source software. The group of experts then brainstormed on how this will possibly affect society, both negative and positive: i.e. more flexible and quicker in terms of innovations (positive), lower level of security when handling data (negative), higher availability of software (positive). The experts were asked this with the aim to list both social, economic and environmental impacts of the trend.

2)In the next round it has been looked away from the original trend, and the primary impact (green circles on Fig. 2) became the level of analysis. How will more flexible and quick innovation impact society? How will lower security when handling data impact society? Again, both positive and negative impacts were listed, and both social, economic and environmental impacts were included.

3)When the secondary impacts are established: Smarter solutions for better analysing, harmful effects for individuals if GDPR data is hacked, malicious users creating bugs i.e. to infect hardware. etc., a last step using the secondary impacts (yellow circles on Fig. 2) as the basis of analysis is made. How will Smarter solutions for better analysing impact society? How will malicious users creating bugs i.e. to infect hardware impact society? Again, both positive and negative impacts were listed, and both social, economic and environmental impacts should be included.

4)Based on the analysis of secondary impacts a third and final circle of tertiary impacts (red circles on Fig. 2) was listed. The brainstorming session ended here with a list of economic, social and environmental impacts on the trend.

Instructions about the methodology was sent out to all participants before the workshop. After the workshop the participants also got a chance to comment on the text and wheels of their own group, as a quality control. One future wheel was developed for each of the four above mentioned areas (OSS, CS, OA, OD), which have then been analysed in relation to society, economy and environment. For presentation reasons and increased readability, we have presented the wheels in excel formats.

The Futures Wheel is mainly concerned with the consequences of a given trend. Hereby not considering that the trend might turn due to potentially negative consequences. Also, it does not consider potential problems in the start-up phase of the new trend. This will further be debated in the discussion section.

## Results

The point of departure for the discussion is that all areas of open science are increasing (in terms of use and sharing), and how a future increase in these areas will impact society, economy and environment in a positive or negative way. The workshop included transport experts within both Open Data, Open Source Software, Open Access and Citizen science.

The experts were both representing research organisations, industry (PTAs, librarians etc.), and transport research umbrella organisations among others. Below are the results from the workshop, covering all four categories.

### Open data

Open data is defined in the Open Definition project as data “that can be freely used, modified, and shared by anyone for any purpose”^50^. Availability and access, reuse and redistribution of data, and universal participation are the key factors of Open data and content^51^. Below are the main findings (Fig. 1) of how increased OD will impact the transport sector and a discussion surrounding the findings.

**Figure 3:**
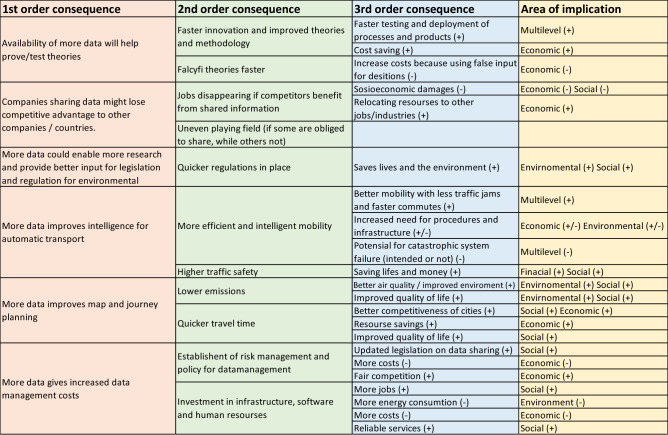
Impacts of open data in the transport sector.

According to the experts, availability of more data will help prove/test theories quicker as a better analysis can be performed with more data available (Fig. 3). This will potentially lead to faster innovation and increased efficiency, e.g. products may enter the market quicker. However, increases in speed and efficiency may also lead to a corresponding increase in errors. In the transport industry (not academia) the availability of more data already leads to changing models with faster testing and faster deployment. Furthermore, processes are increasingly characterized by trial-and-error approaches and on-the-go changes. Quicker innovations also could lead to economic benefits as you spend less time on the tasks at hand.

On the other side, companies sharing data might lose competitive advantage to other companies or countries (Fig. 1). I.e. ship building used to be mainly done in Europe, then it moved to Japan and Korea, before currently China has taken over the main market. The loss of competitive advantage may cause jobs disappearing if competitors benefit from the shared information. The consequences could be quite large, if for example the Port of Rotterdam lost its competitive advantage to Port of Lisbon, then a lot of other industries around the transport industry would also be affected. In terms of data sharing in the transport industry/urban mobility, this would further be amplified by a possible uneven playing field, if some are obliged to share, while others not—causing companies losing contracts. The end consequence could be socioeconomic damages for the businesses and/or countries affected. However, loss of jobs could also cause a relocation of resources, having a positive effect generating more resources for other industries. To avoid losing the competitive advantage, the experts expect development of new business models, again affecting everything down to the types of jobs available. If you must put your data open in the market (i.e. all EU projects demand open sharing of results)—new business models will emerge consequently to deal with this.

Access to more data could potentially also enable more research and provide better input for legislation and regulation for environmental consequences (Fig. 1). I.e. it is easier to show the consequences of environmental data and how it will impact different countries and thereby give better input for regulations both globally and nationally. One of the experts mentioned a specific example from a project where they did not have data from all countries involved, and thereby the process of giving input to the decision-making process was slower. If data was shared, better decisions can be made from the start and faster input to the decision-making process is possible. This would potentially have huge effects on the environment, if you put a regulation in earlier you could save both lives and the environment.

More data could also improve intelligence for automatic transport and makes us less reliant on people (Fig. 1). This will lead to the possibility to increase traffic safety as machines react faster than people. Again, this could lead to saving lives and financial costs. However, it will also have the disadvantage of people losing jobs to automatic processes. Another consequence of more use of automatic transport systems is the potential of catastrophic system failure (intended cyber-attacks or unintended errors). To avoid this there is also an increased need for appropriate procedures and infrastructure for this new landscape. Improved intelligence for automatic transport could also lead to more efficient mobility, but in order to get a better system with less traffic jams, less time commuting to work, it also depends a lot on the strategies put in place by the authorities. Even if you have available open data for all the users in the system, it does not remove cars. However, making the system more efficient could lead to better mobility in combination with the type of policy the city will put in place.

According to the experts, increased use of open data could lead to better journey planning with improved maps and applications (Fig. 1). This could again potentially reduce travel times, giving people an enhanced quality of life since they can allocate travel time into more leisure time. Reducing travel times also reduces costs for the society as travel time could be allocated to extended working hours. All in all, this will be giving the cities a more competitive advantage (economic development). Better journey planning could also give less emission if it leads to reduced travel. This will give us a healthier environment, improved quality of life and also improved health of people.

In terms of data there is a distinction of what type of data we are talking about. Some data, companies are happy to share, and some data sharing will give them a disadvantage as mentioned above. The data has value, depending on whom it is given to. There are two elements—whom you are sharing with and in what type of framework and conditions do you have to share? Should companies openly share, and then everyone has access? Or should you have to ask for access and pay for getting the data? Sharing data entails a transaction cost. The second point is what type of format, and how to put it in a way that's accessible to others? Solving these types of questions will both lead to a need for establishment of policies and risk management related to open data and a need for investments in infrastructure, software and human resources (Fig. 1). This will again increase data management cost. The amount of data available could also lead to trouble if you cannot handle them. Which again leads to increased need for infrastructure—again generating more data management costs. Investments in infrastructure, software and human resources will possibly generate more jobs and more reliable services as a positive outcome, but it will also lead to an increase in energy consumption for the establishment phase, possibly having a negative environmental impact. The establishment of policies and risk management can lead to fair competition and updated legislation on data sharing.

### Open source software

**Figure 4:**
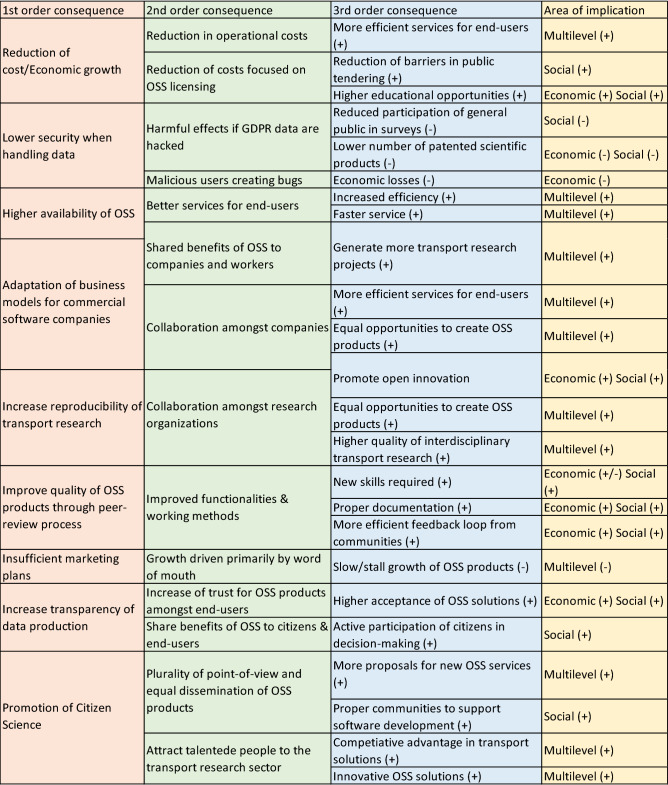
Impacts of open source software in the transport sector.

Open Source Software (OSS) is a software with a source code that anyone can inspect, modify, and enhance. OSS applications have been increasing over the last decades and expanding in new key areas, such as transportation, tourism, health, etc.^22^. Below are the main findings (Fig. 4) of how increased OSS will impact the transport sector and a discussion surrounding the findings.

Investment costs can be divided in two main categories, i.e., the hardware-related expenses (computer units and expendables, specific machinery, etc.) and the software-related expenses (licenses, customer support, maintenance, etc.). Utilization of OSS applications could significantly reduce investment costs as well as costs for operational, maintenance and upgrading purposes by mainly eliminating the cost of licensing of software products. As such, both scientific research communities and private transport industry stakeholders could benefit, and economic growth opportunities could be increased by using OSS. In more detail, OSS products and applications could be patented, reducing the existing barriers in public tendering. In addition, OSS products could provide a higher number of educational opportunities for students/researchers enabling them to capitalize new skills and knowledge.

In this context, an increase in usage of OSS products could provide higher availability of software solutions for the research communities and could also increase the reproducibility of transport research outcomes. Thus, published results could be evaluated to reflect true findings or false positives. This also supports transparency and allows reviewers or other researchers to validate the published results. Additionally, new collaborative schemes between companies and research organizations could be promoted and the awareness and knowledge of OSS benefits towards the public (citizens & end-users) could be increased. New collaborative schemes and public engagement could further support OSS to provide more efficient services, innovative products and tools that could actively engage the general public in decision-making processes for transport research planning or related activities.

Analysing the current tools (e.g., scenario-based optimization, simulation software, modelling applications) for urban transport planning, it is observed that OSS applications and products could promote Citizen Science by achieving a higher involvement of general public and engaging them into the scientific research world. They could offer valuable information and real-time data about the urban transportation network which could result in a competitive and innovative research environment. The engagement of certain communities or citizens could also generate alternative transportation solutions or new transport planning initiatives based on their personal experiences by utilizing and exploiting OSS. Hence, improved quality of OSS products could be also achieved through peer-review processes by testing OSS products for defects. Finally, it is worth mentioning that collaboration among the transport research community and general public could set the basis for creating proper documentation of existing OSS tools.

Since OSS tools can be used by everyone, the steps followed for gaining the final results are freely available offering a more transparent framework that allows a more trustworthy provision and handling process of data to end-users. On the other hand, these lower security standards fail to follow GDPR and avoid cyberattacks and hacking. Mitigation actions are necessary to enhance general public participation in OSS products in an attempt to ensure qualitative data and their participation at a long-term horizon so as to achieve increased patents and profits. Towards an increased use of OSS, private software providers will be forced to properly adapt their business models in order to enhance the competitiveness between the research community and industry. As such, more advanced tools and solutions could be developed offering more effective and efficient tools, applications and solution approaches.

However, no one organisation owns OSS and there is no one to provide sufficient marketing. It is mainly promoted via a “word of mouth” approach that could be insufficient on some occasions. Many OSS initiatives created from public research organizations have failed or delayed reaching the market due to the slow growth of their product or lack of end-users engagement to test their services, even though they are strongly supported by research communities (e.g. Python, R, QGIS, etc.).

### Open access

**Figure 5:**
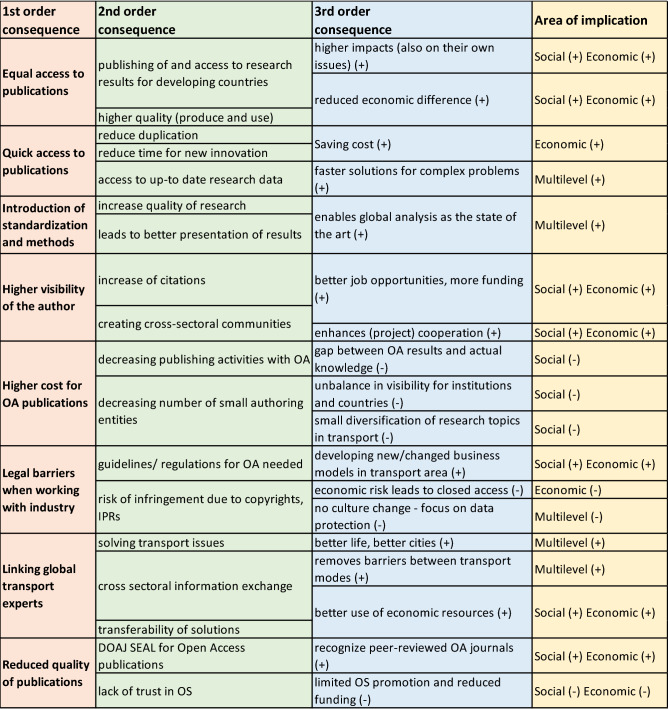
Impacts of open access in the transport sector.

Open Access (OA) is in general known as the process of removing barriers such as price (including subscriptions, licensing fees, pay-per-view fees) and permission (e.g. copyright and licensing restrictions), in order to enable free, online access to full-text information. Below are the main findings (Fig. 5) of how increased OA will impact the transport sector and a discussion surrounding the findings.

Increased usage of Open Access (OA) creates several opportunities as well as exposes the weaknesses which are important to address in order to move towards Open Science (Fig. 5).

Usage of Open Access enables equal access to publications as everybody, independent from the social status, monetary possibilities, country—developed or developing have a possibility to access the research results. This leads to better and broader publishing and using research results concluding higher quality of the research. It could create better awareness in the society by pinpointing strategically important areas where actions need to be taken to reduce economic differences.

Broader use of Open Access in the transport sector can enable stakeholders access to publication in miscellaneous areas and reduce duplications in the database as more information is available and there is no need to “reinvent the wheel”. Having accessible information from other sectors for fast analyses could also potentially increase innovation. The probability that the research data is up-to-date is also higher as it is more used from various stakeholders. Consequently, solutions for complex problems could be found faster and by re-using research data, cost can be saved.

Open Access is a challenge in the transport sector whereas the amount and complexity of data needs to be accommodated. In order to enable Open Access in the sector and to broaden the use, introduction of standardization and methods are important factors. Therefore, guidelines and recommendations at the European level need to be developed for Open Access in the transport sector and supportive infrastructure need to put in place. This would possibly increase the quality of research and leads to better presentation of the results, paving the way for an analysis of issues or challenges on a global level as the state of the art.

A well-known factor is that Open Access is increasing the visibility of the publication as the publications are freely available to a broader audience, which also gives higher visibility for the author. This could be measured, using the growth of citations, making the author more popular and famous—and therefore supporting the researcher's scientific career. This again could trigger better job opportunities resulting in more funding. On the other hand, the creation of the new international communities could lead to more projects, enhancing cooperation in Horizon Europe for example and therefore enable international funding.

The increasing request for free-of-charge access to full-text information affects the pricing formula for journal publications. Raising Article Publishing Charges (APC) leads to higher cost for OA publications on the author side. Due to limited budgets for publishing the overall publishing activities with OA could decrease, which could lead to a gap between OA results and the actual knowledge available in the research domain. This trend could be intensified by the fact that small authoring entities might not be in the position to pay higher APCs so that OA publication would represent less diversification of the research topics in transport. For example, institutes from third world countries with lower research budgets can only publish their results at high cost and thus lose opportunities for perception. In the most extreme case, this can lead to an even stronger unbalance in the visibility of institutions and countries.

In the context of publishing results in general, there are several legal barriers when working with industry partners, which gets even more critical if OA is involved. There is the risk of infringement due to copyrights and IPRs leading to a focus on data protection, in case of doubt, and preventing a cultural change. Furthermore, even if publication is an option, «closed access» could be preferred to minimize a possible economic risk in the highly competitive transport area.

To pre-empt those consequences by legal barriers, clear guidelines and regulations for OA in transport are needed. It would introduce a framework which allows to develop new/changed business models in the transport area, based on sharing information via OA publications.

A free-of-charge, online access to full-text information provides new opportunities to link global transport experts. It allows a cross-sectoral information exchange due to removed pay-walls and easier accessibility. An example could be that scientists from the field of aeronautics can benefit from similar problems on fluid dynamics in the field of shipping. Barriers between the transport modes can be lowered or even fully removed. It could lead to a transferability of solutions beyond transport modes, so that beside the increasing information exchange existing economic resources can be used better/more effectively. Profiting from the linking of global transport export, there is a higher chance to solve transport issues in a common approach to improve cities, e.g. challenges in interaction of different transport modes in urban transport scenarios, and consequently life aspects.

There is the fear that an increased use of OA would lead to a reduced quality of the publications. If confirmed, the risk increases that the transport community will lose the trust in Open Science in general and the number of OS activities could decrease. That leads to limited OS promotion in general, ending in reduced funding for transport research as more funding agencies indicate OA as a funding condition. To prevent a quality reduction of publications, the DOAJ Seal was established which is awarded to journals that demonstrate best practice in open access publishing and simplifies to recognize peer-reviewed OA journals.

### Citizen science

**Figure 6:**
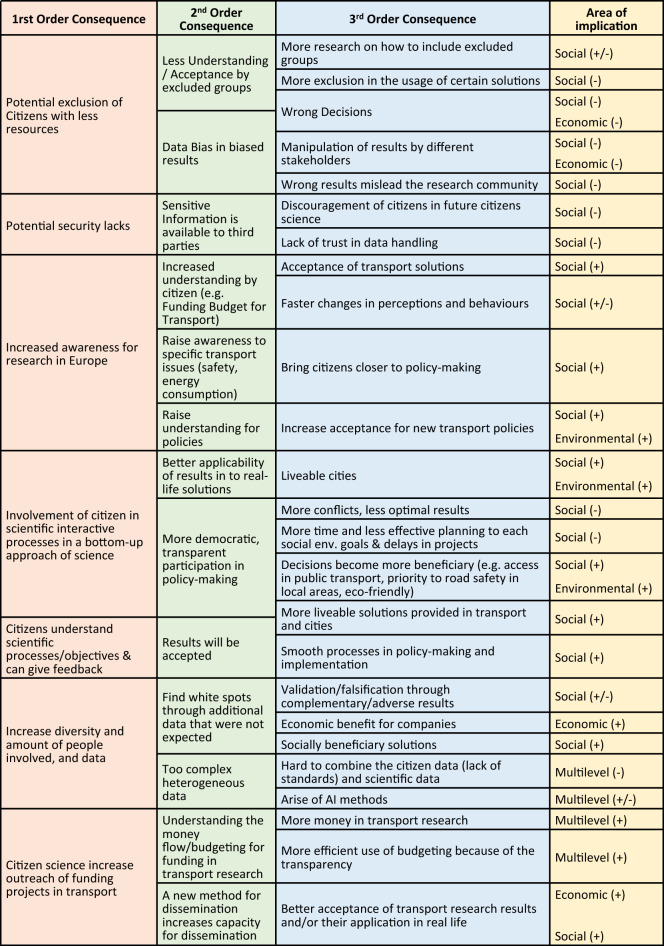
Impacts of citizen science in the transport sector.

Citizen science is a way to connect professional scientists and the public in which the public directly contributes to the production of knowledge, sometimes restricted to data collection or simple analysis but also involving more substantial activities. Citizen science has been on the increase since the 1990s and it is also known as community science or described as public participation in scientific research^52^. Below are the main findings (Fig. 6) of how increased CS will impact the transport sector and a discussion surrounding the findings.

The practice of citizen science fosters the involvement of citizens in scientific interactive processes in a bottom-up approach of science. According to the experts, this could improve the applicability of results in the real life of citizens, e.g. through simplifying sustainable transport solutions by the help of the input of citizens testing prototypes in real-life laboratories. Thereby, citizens can contribute to make their own city or municipality more liveable. The involvement and participation could lead to more transparency of policy decisions and democratizes the process. A plausible consequence would be that the solutions could become more beneficial to citizens since they co-create, co-implement and/or co-evaluate.

The practice of citizen science fosters the involvement of citizens in scientific interactive processes in a bottom-up approach of science. This could improve the applicability of results in the real life of citizens, e.g. through simplifying sustainable transport solutions by the help of the input of citizens testing prototypes in real-life laboratories. Thereby, citizens can contribute to make their own city or municipality more liveable. The involvement and participation can lead to more transparency of policy decisions and democratizes the process. A plausible consequence would be that the solutions could become more beneficial to citizens since they co-create, co-implement and/or co-evaluate solutions making those safer and eco-friendlier. A negative impact would be that the democratization slows down decision processes e.g. in city planning and thereby slows down the implementation of new transport solutions. Further, through participation conflicts can occur leading to less optimal results through compromises. In contrast, results could also be optimized through constructive decisions leading to more liveable cities for all.

On the one hand, the involvement of citizens in science can increase the diversity of people providing a coherent data set to the research project. Science can benefit from this because they can detect white spots through additional data they would not have gained if they had selected only certain target groups. This could potentially impact the validation or falsification practice in research through the complementary or adverse results and thereby work as an accelerator for new research to improve findings. Above that, white spots in research can also be market white spots companies could exploit. The society could benefit through better science because new transport solutions can be designed in a way to solve the newly identified problems. On the other hand, data sets gained through citizen science can be complex or heterogeneous. This could impact the possibilities to combine data with other existing data sets coming from other citizens science due to a lack in standards or with scientific data due to quality differences. To cope with the complexity of data, AI methods could be further developed consequently.

The practice of citizen science can potentially exclude citizens with less resources, e.g. professionals or families with young children having less time in evening hours, etc. The exclusion could result in less or no understanding or acceptance of research outcomes. This could potentially also lead to wrong political decisions. Following that, excluded groups could not benefit from differently designed transport solutions being not relevant to them or designed in a way they exclude groups, e.g. with special needs. Consequently, more research on how to address and include diverse groups must be undertaken. The occurring data bias and biased results could mislead the research community trying to reuse open data sets. Further, stakeholders could use the biased data to prove solutions in agenda-setting and lobby work.

Another point addressed by the experts is that potential security lacks could make sensitive information available to third parties leading on the one hand to the discouragement of citizens in taking part in citizen science projects. On the other hand, it could heat up the debate on the security of data handling in Europe.

Taking part in citizen science can raise the awareness for (funded) research activities in Europe, so citizens can benefit from a greater transparency, e.g. they can gain a better understanding on research results or the budget flows for research and innovation in transport. This could possibly impact their acceptance as well as their perception of new transport solutions and even their mobility behaviour itself. Secondly, attending citizen science projects can help to raise the awareness of citizens for specific transport issues, such as safety, energy consumption, design of new mobility solutions, etc. This allows citizens to contribute to policy making. At last, the increased awareness for research in Europe can help citizens to understand and thereby accept new policies in transport, even if this means uncomfortable regulations for them that should tackle ecologic challenges for instance.

Citizen science in practice increases the outreach of funded projects in transport research. The understanding of money flows and budgets and outcomes of funding in transport research can thereby be raised. This could lead to the demand of citizens for more public research in how transport is funded. Citizen science can even be used as a means of dissemination leading to higher acceptance of transport research results and their application in transport.

## Discussion

Even though the groups have been divided into four different themes in the workshop, most of the outputs are true for all areas of OS even though the focus was slightly different in the different working groups.

The results from the workshop gave more insight to social and economic impacts, as compared with environmental concerns as we can see on Figs. 3, 4, 5, 6. However, the environment specific impacts could potentially have large consequences: lower emission, quicker regulations, and faster acceptance of transport politics from the general public are crucial to meet the UN goals of reducing emission rates. Also, the multilevel impacts cover all the three areas (social, economic, environmental). Some of the inputs are placed in the area where it has the largest impacts, however most impacts will affect all other areas to some extent.

Supporting and confirming former sources^8,17,19,29,34,36,39^ we found a lot of similar general implications of OS in the future:

Open Science may have the possibility to create more equality due to better access of research for more people/countries/institutions through open access publishing, creating equal competition through new policies for data management and creating equal opportunities through free use of OSS. We also found that open Science might increase efficiency, through less time spent on data collection/duplicating work, better efficiency for end-users due to reduction in operational cost and solving complex problems faster due to access to more data.

Economically there might be benefits through more efficient budgeting because of transparency, less time spent on duplication of work and collecting data, improved knowledge exchange and increased funding due to better marketing of research. Additionally, Open Science may lead to innovation and new technology. First of all, economic savings could potentially free a lot of resources that can be relocated to innovation activities. Secondly, the increased availability of data could lead to potentially faster innovation and arising use of AI technology to handle the amounts of data. In terms of citizens participation, it will bring citizens closer to policy making processes and hence potentially raise the awareness in the general public, simplifying the process of implementing new policies.

In addition to reproduce some of the same conclusions as former studies the Futures Wheel workshop identified some transport specific implications of open science:

Lower emission improving Quality of Life and air quality due to better planning of transport systems are one of the positive environmental impacts specific to the transport sector. This may also be improved through an increased awareness causing an increased acceptance of transport solutions/politics and faster changes in perceptions and behaviour i.e. important to change travel behaviour to reduce emission.

Faster travel time increasing quality of life, saving costs and improving cities competitive advantage is another possible transport specific output of Open Science. The possibility to improve AI systems would potentially increase traffic safety, improve mobility services and improve commuting. However, AI systems also have some unknown risks.

The competitive advantage of participating in Open Science can be seen both through better marketing of research, being able to relocate resources to other tasks and finding new white spots. It was mentioned that OS has the potential to attract more money into transport research, generate new projects and attract talented people to transport research.

Most former studies and the general attitudes towards Open Science are positive, and in this study, we also found more positive than negative impacts. However, we did establish some negative impacts that is important to have in mind for OS to become as sustainable as possible.

First, job loss due to loss of competitive advantage and increased use of artificial intelligence is one potential problem. Open Science will also create new type of risks—hacking & malicious users creating software bugs and potential system failures with larger unknown consequences are just two examples. This bring us over to the next negative—where Open Science in the short term might increase cost due to extra infrastructure needs and repair costs for malicious users creating software bugs. New infrastructure might also generate cost in terms of energy consumption. Another possible negative effect is that Open Science might increase decision making based on ‘wrong data’ due to low quality data, data bias or lack of knowledge of how to use the data (as data might be used by other people not trained in using the available data). Open Science might also increase conflicts due to more participants involved in research. This again might cause time delays. There is also the problematic aspect of increasing the equality gap due to decision making on biased data—hence where one is excluding already vulnerable groups from citizen science.

To address the negative impacts of Open Science also have great practical implications on how to carry on the development of Open Science in transport further. Identifying possible negative outcomes is the first step in a risk analysis—making it possible to prevent these outcomes from happening. The paper demonstrates the importance of including negative impacts of this phenomenon, as most former literature and attitudes in the transport sector has a generally been positive. Next steps would be to do a risk assessment of each individual implications mentioned—and to analysis the probability of each consequence and hence create a plan of mitigating actions in or der to avoid the negative consequences.

Using the Futures Wheel as a methodological tool to answer what consequences Open Science will potentially have on the society revealed potential weaknesses of the model. Firstly, applying the model on this particular research question, the output fails to take into consideration that the negative consequences might stop the trend of “increased use”. The negative effects of losing competitive advantage, costs of infrastructure and increased conflicts might reduce the potential of Open Science continuing to increase. Secondly, a more general problem with the model is the fact that the findings will very much depend upon what kind of experts are included in the workshops. This problem is however possible to reduce when putting together the workshop—making sure you have experts from different fields of expertise. As we have looked upon Open Science from four different angles, we have further reduced this problem. However, we still seem to have gathered more input on social and economic impacts, which may be a reflection of the field of expertise of the experts. On the positive note, as this study confirms former findings on open science in general, the methodology seems to be an efficient alternative to the more time-consuming Delfi-methodology.

Even though there might be problematic aspects linked to Open Science, shedding light on these aspects will also help us implement it in a more sustainable way. To deal with this new emerging way of sharing data and research, as mentioned, proper risk management systems are important to establish to avoid any negative impacts. A lot of the negative impacts are possible to avoid if planned for. In terms of job loss due to AIs—this might free recourses for other jobs. I.e. improve the amount of time personnel has to offer service to customers. This way it is possible to turn around the potential negative effects to positive ones. As this study has identified the most important problematic areas of implementing Open Science in the transport sector, the results may be used by several different actors in the transport sector.

## Conclusions

Open Science may generate a lot of positive impacts on both the economic, social and environmental arena. For the transport sector especially better journey planning, improvements in AI systems and easier acceptance of political decisions through citizen science are important. To avoid the negative effects on loss of competitive disadvantage, some of the experts also focused on the advantage of finding white spots and better use of resource.

As we have seen there are potential negative outcomes in the same areas as there are positive. Increased OS can both cause economic losses or economic gains, both lower emission and increase energy consumption, both improve equality and create more exclusion of vulnerable groups and both increase efficiency and reduce it. Loss of competitive advantage for some groups is important to consider in order to prevent potential losses, but also to understand why some nations, industries and individuals are not in favor of sharing. If we know the negative outcomes it is much easier to put in place strategies that are sustainable for a broader stakeholder group, which also increase the probability of taking advantage of all the positive impacts of Open Science. More research is needed on the individual impacts in order to create specific strategies for sustainably implement OS. Also, quantitative analysis like cost-benefits analysis, are needed to establish the actual numbers for each impact.

## Data Availability

All data generated and analyzed during this study are included in this published article in the tables, except for the sound files which were the background for creating the tables. The sound files recorded in the workshops were deleted at the end of the project due to GDPR.
